# Electromagnetic Field Exposure in Kindergarten Children: Responsive Health Risk Concern

**DOI:** 10.3389/fped.2021.694407

**Published:** 2021-07-05

**Authors:** Shiva Raj Acharya, Yong Chul Shin, Deog Hwan Moon, Sandip Pahari

**Affiliations:** ^1^Graduate School of Public Health, Busan Medical Campus, Inje University, Busan, South Korea; ^2^Department of Occupational Health and Safety, Inje University, Busan, South Korea; ^3^Department of Public Health, Pokhara University, Pokhara, Nepal

**Keywords:** electromagnetic field, transmission line, exposure, children, risk

## Abstract

Long-term exposure to physical agents can be detrimental to children due to their vulnerability. This study aimed to assess and compare the electromagnetic field (EMF) exposure level around the kindergartens from the underground transmission line (UGTL). We investigated randomly selected 24 kindergartens based on the location of the UGTL. The EMF emission levels were measured using an EMDEX II (Electric and Magnetic Digital Exposure Meter). The maximum mean value of the EMF emission level was 13.5 mG around the kindergartens and 17.7 mG from the point of UGTL to kindergartens. EMF emission level around the kindergartens was significantly associated with the location of the UGTL (*t* = −7.35, *P* < 0.001). These estimates are not trivial, as long-term exposure to EMF among kindergarten children can lead to different health problems. Routine monitoring of EMF emission levels is recommended including the awareness of EMF exposure to public citizens.

## Introduction

Electromagnetic fields (EMFs) are a form of radiation energy associated with the modern use of electrical power. Sensitive areas like schools, kindergartens, hospital, and other public facilities have been of great concerns of various studies. EMF exposure can lead to environmental impacts (1). In city areas, transmission lines that are distributed underground still can pose negative consequences to the general public. Kindergartens, schools, and hospitals are more sensitive to EMF exposure. Exposure to EMF has been correlated with the occurrence in humans, including infants, of potential adverse biological and health effects (2). A variety of studies, however, have not identified statistically significant associations between exposure to EMF and health risks. Although numerous studies have been performed to assess environmental EMF exposures, they are mainly concentrated on assessing exposure in adult populations. Restricted information is available on EMF exposure levels and their related settings in kindergartens and schools (3, 4).

While most of us over the years have probably heard rumblings of the possibilities of negative health effects attributed to high levels of exposure to EMFs, little definitive word has reached the mainstream stamping it as a legitimate concern. The concern about exposure to EMF has developed because of the number of epidemiological studies (5). EMF exposure can pose impacts on the public and the environment. Mostly in city areas, the transmission lines are distributed and constructed underground to minimize the exposure limits. Children's nervous system is more susceptible than that of adolescents to the effects of EMF exposure. Even though many of us have arguably noticed unconfirmed reports of the possibilities of negative health impacts linked with EMF exposure over the years, no particular set has gone mainstream embroidering it as a real issue (6, 7).

This paper is a short research commentary article based on findings from the assessment and comparison of the EMF emission level from the underground transmission line (UGTL) around kindergartens. The study focuses on the EMF emission level assessment in kindergartens located around the UGTL to address the immediate concern of EMF exposure among children.

## Methods

**Figure 1:**
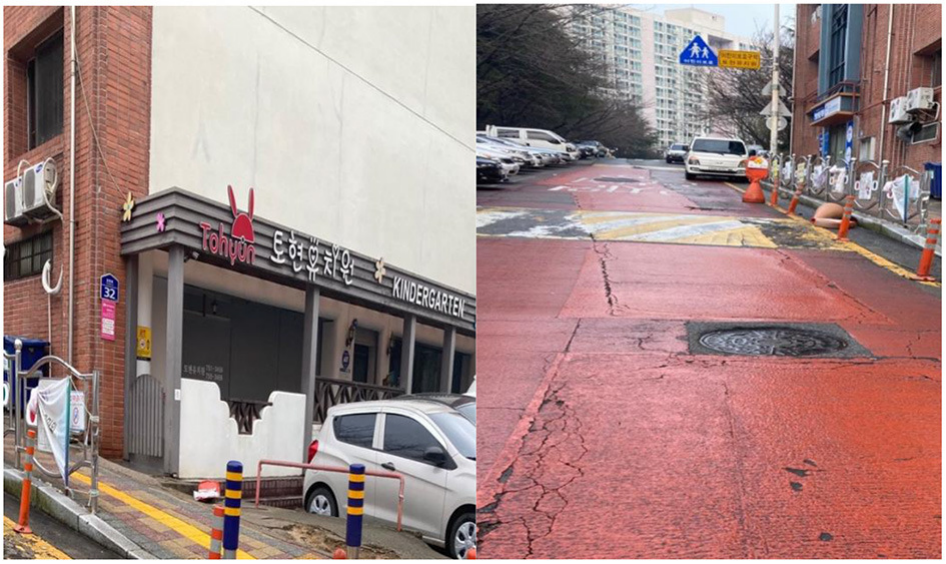
Spot measurement around kindergarten and from the underground transmission line (UGTL) to kindergarten.

The EMF emission levels in kindergartens were measured based on the location of the UGTL in December 2020. This study includes 24 kindergartens across Busan, South Korea (12 kindergartens located near UGTL and 12 kindergartens without UGTL). The UGTL locations and kindergartens in Busan City were accessed from Busan Korea Electric Corporation and Ministry of Education, Korea, respectively. Then, 24 kindergartens were sampled randomly from the list of kindergartens based on the UGTL location. We calculated the EMF emission levels with two measurement techniques as shown in Figure 1: (i) EMF measurement around the kindergartens and (ii) EMF measurement from the point of the UGTL to kindergartens. The emission levels of EMF were measured using EMDEX II (Electric and Magnetic Digital Exposure Meter). At 0.5, 1, and 1.5 m from ground level, we monitored the EMF emission levels. The data were analyzed using the Statistical Package for Social Science (SPSS) version 23. To identify the association of EMF emission and UGTL, a simple linear regression analysis was performed.

## Results and Discussion

Table 1 shows the variation in EMF emission levels in the selected kindergartens. The maximum mean value of 13.5 mG was monitored around kindergarten, and 17.7 mG was measured from the UGTL to kindergarten, which is a relatively high emission level. Based on the UGTL, EMF level was found to be very low (below 1 mG) in kindergartens that were not located around transmission lines as compared with kindergartens with transmission lines nearby. EMF emission level around the kindergartens was found to be statistically significant with UGTL location (*P* < 0.01).

**Table 1:** EMF emission level in selected kindergartens.

**Kindergarten**	**Kindergarten with UGTL**				**Kindergarten without UGTL**
	**Mean EMF level around kindergarten (mG)**		**Mean EMF level from UGTL point to kindergarten (mG)**		**Mean EMF value around kindergarten (mG)**
1	11.2		13.9		0.3
2	6.2		4.7		0.1
3	5.1		8.5		0.2
4	13.5		17.7		0.5
5	7.3		5.2		0.2
6	7.1		5.9		0.2
7	4.9		9.2		0.2
8	4.2		7.1		0.8
9	5.2		11.1		0.4
10	6.2		11.3		0.5
11	5.6		11.1		0.2
12	4.9		4.3		0.3
**Variable**	**EMF emission level around kindergarten**				
	**B**	**SE**	**β**	***t***	***P***
Constant	15.70	1.47		10.62	0.000
UGTL location	−7.69	1.04	−0.78	−7.35	0.000

*R^2^ = 0.61, adjusted R^2^ = 0.60, F = 54.09, P < 0.01*.

*EMF, electromagnetic field; UGTL, underground transmission line*.

EMF emission levels in kindergartens located along the transmission line were found to be lower than those of the international (<2,000 mG) and Korean (<833 mG) guidelines (5, 8). According to the WHO research findings, studies had shown that long-term exposure to EMF may be a possible risk factor for childhood leukemia (9). To find out whether there is a causal correlation, numerous studies have comprehensively studied power lines and cancers. Previous studies showed that power-line-generated EMFs have frequencies that are too low to impact living cells or damage DNA. There is insufficient evidence available to establish a justification for setting exposure limits in regard to the possible long-term consequences of exposure (e.g., leukemia) (3, 9). The International Commission on Non-Ionizing Radiation Protection (ICNIRP) recommendations for reducing EMF effects on humans including children have been adopted worldwide (10). They primarily guard against the short-term health effects of exposure to EMFs. EMFs from power lines, through electric fields induced in the body, may cause significant changes in the biological system (5–11). Kindergartens are considered to be highly exposed, and long-term EMF exposure possibly leads to health risks especially on children from kindergartens (7, 12–14). Different methods have been recommended by some countries to restrict EMF exposure in younger children. In South Korea, they also embraced the ICNIRP recommendations to limit the sensitivity of EMF exposure, but still, public citizens and parents are not highly concerned about the negative impacts of EMF exposure on children. To restrict public exposure to power-frequency EMF, scientific criteria have been established such that induced currents are below those that occur naturally in the body (3, 5). While the guidelines and reports cover different causes, they do not explicitly cover possible health risks associated with EMF exposure on a long-term basis. In our investigation, EMF emission levels on selected kindergartens were found to be significantly higher compared with those in other kindergartens not located near UGTL, supported by the study conducted in kindergartens to assess the RF-EMF emission levels (1). In certain situations, if the EMF level is high around the kindergartens, there might be some other electrical sources that may affect the EMF value if there is no UGTL nearby. Our preliminary findings have implications for the design of larger studies and the improvement of EMF-related policies in South Korea and other nations. Besides, EMF exposure effects on children are not well-established, children's susceptibility to EMF exposure should be lowered, and safety precaution standards should be enforced. Furthermore, potential hazards from EMF exposure should be studied more accurately to develop appropriate public policy for the protection of the children's health.

## Conclusion

This article provides the ideal concept for conducting the personal EMF exposure assessment from UGTL among the children for future EMF-related epidemiological studies. It is not appropriate to ignore the fact that EMFs are potentially carcinogenic to children. Proper installation solutions should be implemented for the minimization of the EMF level around the public sensitive areas with routine monitoring.

## Data Availability Statement

The original contributions presented in the study are included in the article/supplementary material, further inquiries can be directed to the corresponding author.

## Ethics Statement

The studies involving human participants were reviewed and approved by Ethical Review Board of Inje University, South Korea. The patients/participants provided their written informed consent to participate in this study.

## Author Contributions

SA interpreted the study information and wrote the manuscript. YS and DM performed discussion and analysis. SP assisted in literature discussion and analysis. All authors contributed to the article and approved the submitted version.

## Conflict of Interest

The authors declare that the research was conducted in the absence of any commercial or financial relationships that could be construed as a potential conflict of interest.
